# Smart forecasting of artifacts in contrast-enhanced breast MRI before contrast agent administration

**DOI:** 10.1007/s00330-023-10469-7

**Published:** 2023-12-15

**Authors:** Andrzej Liebert, Badhan K. Das, Lorenz A. Kapsner, Jessica Eberle, Dominika Skwierawska, Lukas Folle, Hannes Schreiter, Frederik B. Laun, Sabine Ohlmeyer, Michael Uder, Evelyn Wenkel, Sebastian Bickelhaupt

**Affiliations:** 1 Institute of Radiology, Universitätsklinikum Erlangen, Friedrich-Alexander-Universität Erlangen-Nürnberg (FAU), Erlangen, Germany; 2 Medical Center for Information and Communication Technology, Universitätsklinikum Erlangen, Erlangen, Germany; 3 Pattern Recognition Lab, Friedrich-Alexander-Universität Erlangen-Nürnberg (FAU), Erlangen, Germany; 4 Medizinische Fakultät, Friedrich-Alexander-Universität Erlangen-Nürnberg (FAU), Erlangen, Germany; 5 Radiologie München, München, Germany; 6 German Cancer Research Center (DKFZ), Heidelberg, Germany

**Keywords:** Magnetic resonance imaging, Breast, Forecasting, Artifact, Neural networks (computer)

## Abstract

**Objectives:**

To evaluate whether artifacts on contrast-enhanced (CE) breast MRI maximum intensity projections (MIPs) might already be forecast before gadolinium-based contrast agent (GBCA) administration during an ongoing examination by analyzing the unenhanced T1-weighted images acquired before the GBCA injection.

**Materials and methods:**

This IRB-approved retrospective analysis consisted of *n* = 2884 breast CE MRI examinations after intravenous administration of GBCA, acquired with *n* = 4 different MRI devices at different field strengths (1.5 T/3 T) during clinical routine. CE-derived subtraction MIPs were used to conduct a multi-class multi-reader evaluation of the presence and severity of artifacts with three independent readers. An ensemble classifier (EC) of five DenseNet models was used to predict artifacts for the post-contrast subtraction MIPs, giving as the input source only the pre-contrast T1-weighted sequence. Thus, the acquisition directly preceded the GBCA injection. The area under ROC (AuROC) and diagnostics accuracy scores were used to assess the performance of the neural network in an independent holdout test set (*n* = 285).

**Results:**

After majority voting, potentially significant artifacts were detected in 53.6% (*n* = 1521) of all breast MRI examinations (age 49.6 ± 12.6 years). In the holdout test set (mean age 49.7 ± 11.8 years), at a specificity level of 89%, the EC could forecast around one-third of artifacts (sensitivity 31%) before GBCA administration, with an AuROC = 0.66.

**Conclusion:**

This study demonstrates the capability of a neural network to forecast the occurrence of artifacts on CE subtraction data before the GBCA administration. If confirmed in larger studies, this might enable a workflow-blended approach to prevent breast MRI artifacts by implementing in-scan personalized predictive algorithms.

**Clinical relevance statement:**

Some artifacts in contrast-enhanced breast MRI maximum intensity projections might be predictable before gadolinium-based contrast agent injection using a neural network.

**Key Points:**

*• Potentially significant artifacts can be observed in a relevant proportion of breast MRI subtraction sequences after gadolinium-based contrast agent administration (GBCA).*

*• Forecasting the occurrence of such artifacts in subtraction maximum intensity projections before GBCA administration for individual patients was feasible at 89% specificity, which allowed correctly predicting one in three future artifacts.*

*• Further research is necessary to investigate the clinical value of such smart personalized imaging approaches.*

**Supplementary Information:**

The online version contains supplementary material available at 10.1007/s00330-023-10469-7.

## Introduction

Breast MRI examinations in clinical routine are commonly performed using a multiparametric MR acquisition protocol. This commonly combines non-contrast-enhanced (non-CE) morphologic and diffusion-weighted imaging (DWI) sequences with pivotal dynamic contrast-enhanced (DCE) T1-weighted sequences (T1w) [1]. The latter imaging is acquired before and repeatedly after the administration of intravenous gadolinium-based contrast agents (GBCAs), allowing for the depiction of alterations in the local perfusion of the breast tissue as a key feature in helping to detect breast cancer [2]. To improve the visualization of perfusion alterations by radiologists, the T1w-DCE sequences are commonly processed to derive so-called subtraction series: the initial T1w acquisition before contrast agent administration is subtracted from the data acquired after contrast agent administration, with the remaining image information containing solely perfusion information. With the increasing interest in incorporating breast MRI in screening settings [3, 4], abbreviated protocols that use maximum intensity projections (MIPs) have raised attention. A MIP image is a 2D representation of a volume, presenting the maximum intensity value in a volume along a certain axis. MIPs allow for a fast initial assessment of suspicious lesions and have thus emerged as a possible initial stratification point for deciding on the presence or absence of a lesion and whether further clarification or characterization using the full set of slices is needed. This pivotal position of MIPs in abbreviated breast MRI approaches highlights the demand for a reliable and artifact-free image quality, which can easily be impeded, e.g., by a subtle motion of the patient during the acquisition blurring the image. However, imaging artifacts are described in many breast MRI examinations [5–9]. As such, the detection of artifact DCE acquisitions is an important part of quality control and allows feedback for the radiologist about regions that might need dedicated attention during reading or indicate the necessity to repeat the examination. Recently, our group has shown that performing such quality assurance–related tasks using convolutional neural networks (CNN) is possible [6]. CNNs are a type of deep learning model specifically designed to process image data by automatically and adaptively learning spatial hierarchies of features [10]. Through this process, CNNs can distinguish between normal and abnormal patterns in the image, enabling the identification and localization of artifacts, for example [6, 11–15]. However, such algorithms can only detect the artifacts after they have occurred, potentially making some acquisition unusable. As such, it would be advantageous if the occurrence of artifacts apparent after the GBCA administration could be prevented in advance. Forecasting the occurrence of such artifacts before the injection of GBCA would allow intervening in the ongoing MRI examination, e.g., by advising the patient to lie still or repeating the unenhanced sequence that precedes GBCA administration and is used for processing the subtraction series.

Therefore, we investigated the capability of a neural network to predict artifacts visible in breast MRI CE subtraction series before the injection of GBCA. We did this by analyzing the non-CE T1w acquisitions preceding contrast agent administration, envisioning a clinically meaningful intervention point for improving breast MRI during ongoing examinations.

## Materials and methods

### Study sample and ethics approval

This IRB-approved, retrospective study was performed using breast MRI examinations performed as part of clinical routine between 2015 and 2020. The inclusion criteria for the patients were clinically indicated breast MRI examinations and the examination being performed with a full diagnostic breast MRI protocol including a DCE imaging series after GBCA administration. The exclusion criteria were male patients and missing DCE acquisitions. Additionally, we excluded examinations from one MRI system on which fewer than 10 examinations were performed during the investigated time. The study cohort was divided into an independent holdout test (*n* = 285 MRI examinations) set and a main training and validation set (*n* = 2559) at 10% and 90% cutoff of the whole data. During this division, it was ensured that the distribution of the mean of the Likert-scale labels given by the three readers was equal in the two sets.

**Fig. 1:**
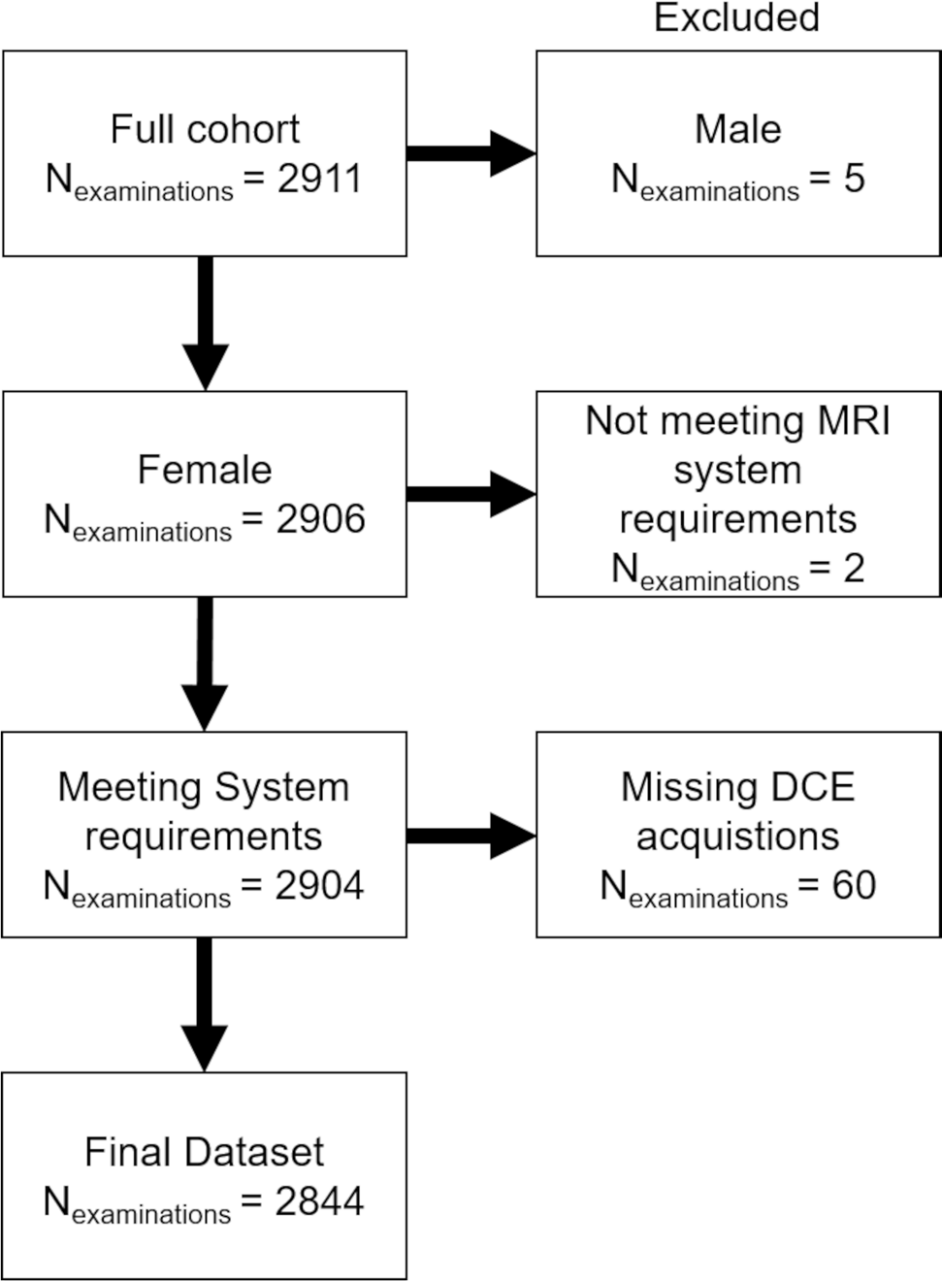
Overview of the *n* = 2844 MRI examinations included in the study. Out of the *n* = 2911 MRI examinations, datasets were excluded due to including male patients (*n* = 5), not meeting MRI system requirements (referring to two examinations being performed at a 1.5-T Sola MRI), and examinations being performed without contrast-enhanced acquisitions (*n* = 60)

Additionally, from the test set, a subset of *n* = 100 consecutive patients was identified for the evaluation of the artifact origin as described in the “Artifact origin evaluation” section. The final study flow diagram is presented in Fig. 1.

Parts of the final patient population (*n* = 2265 and *n* = 1309) were included in previous studies by our group, dealing with the detection of artifacts in CE MIPs after the acquisition [6] and the detection of artifacts in MIPs of high *b*-value DWI [11]. Compared to these, this study focuses on the development of an algorithm focused on the prediction of an artifact before it occurs using just the pre-injection T1w acquisition of the CE MIP.

### MRI protocol

Breast imaging examinations were performed using four different clinical routine MRI devices from a single vendor (Siemens Healthineers) at 1.5 T (Magnetom Aera, Magnetom Avanto) and 3 T (Magnetom Skyra, Magnetom Vida) as previously described [6]. The clinical routine breast MRI protocol consists of a multiparametric protocol including non-CE morphologic (T1-weighted, T2-weighted), DWI, and T1-weighted DCE sequences before and after intravenous GBCA administration. A detailed overview of the MRI parameters from the data analyzed as part of this study (T1-weighted sequences) is shown in Table 1.

**Table 1:** Sequence details for the T1-weighted sequence acquired

Field strength	In-plane matrix size (XxY)	In-plane pixel spacing (mm)	Slice thickness (mm)	TR (ms)	TE (ms)	*N* averages	Additive information
1.5 T	384 × 323–448 × 394	0.76 × 0.76–1.12 × 1.12	1.5–2.1	6.49–8.32	2.39–4.78	1	Acquired before and after GBCA (gadobutrol, Bayer, Leverkusen, Germany), 0.1 mmol/kg/body weight, injection speed 2 ml/s
3 T	256 × 174–448 × 470	0.49 × 0.49–1.0 × 1.0	1.5–1.9	4.13–6.18	1.73–2.46	1	

### Data processing for the reader study

All data were retrieved from the routine clinical picture archiving and communication system of the University Hospital Erlangen using the open-source RSNA CTP pipeline. For the assessment of artifacts, MIPs of the second-phase subtraction series were created. Detailed information on this data processing is given in the Supplement.

### Visual artifact assessment

**Fig. 2:**
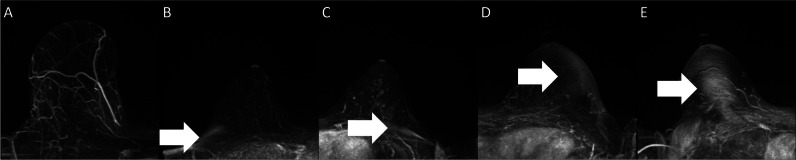
Example of artifact classifications given in dynamic contrast-enhanced (DCE) subtraction maximum intensity projections (MIPs). **A** No artifact at all (score 1); **B** minor artifacts but without the potential to impede the assessment (score 2); **C** moderate artifacts, potentially impairing the visual assessment (score 3); **D** pronounced artifacts (score 4); **E** highly significant artifacts (score 5)

All processed CE subtraction MIPs of the left and right breasts were read regarding the presence and severity of image artifacts by three independent readers reflecting varying degrees of experience in breast MRI (S.B., radiologist, > 10 years of experience in radiology; J.E., medical research assistant, 2 years of experience in MRI; D.S., research assistant, < 1 year of experience in MRI). Reading was performed using a 5-point Likert-like scale: 1—no artifact at all; 2—minor artifacts, without the potential to impede the assessment; 3—moderate artifacts, potentially impairing the visual assessment; 4—pronounced artifacts; and 5—highly significant artifacts. An overview of the different scores attributed to the CE subtraction MIPs is given in Fig. 2.

The rating on the presence of artifacts was performed independent of whether artifacts detected on the image factually covered a significant breast lesion in the individual patient. However, artifacts classified as pronounced or highly significant (score values of 4 and 5) were requested to have the potential to clinically mask a significant finding on the image. To create examination-level labels, the highest artifact values from the left and right breasts were chosen. Additionally, the examination-level artifact scores were binarized, with the scoring labels of 1, 2, and 3 considered “non-significant artifacts” (class 0) and the scoring labels of 4 and 5 considered “potentially significant artifacts” (class 1) for further processing. After this binarization process, final examination classes (class 0 or class 1) were computed from the three readings using a majority vote approach. The binarization was chosen to provide insight into clinically applicable workflow-immersive approaches in which a binarized recommendation would provide greater clarity on whether to repeat an acquisition than purely providing ratings on visual multiclass scales.

### Artifact origin evaluation

To investigate the origin of the identified artifacts, consecutive *n* = 100 cases from the test dataset were additively evaluated by a single reader (S.B., radiologist, > 10 years of experience). The reader performed a side-by-side evaluation by examining the original T1w acquisition, the CE T1w acquisition, the CE T1w subtraction images, and the MIP of the CE subtraction images. During the evaluation, the images were inspected for movement-induced blurring in the pre-contrast or post-contrast volume or changes in breast position between the two volumes. The volumes were also inspected for the presence of skin enhancement (not necessarily accompanied by pathologic thickening) or implants demonstrating subtle enhancing lining around the capsule without fulfilling the diagnostic criteria for capsule fibrosis.

### Neural network architecture setup and training

**Fig. 3:**
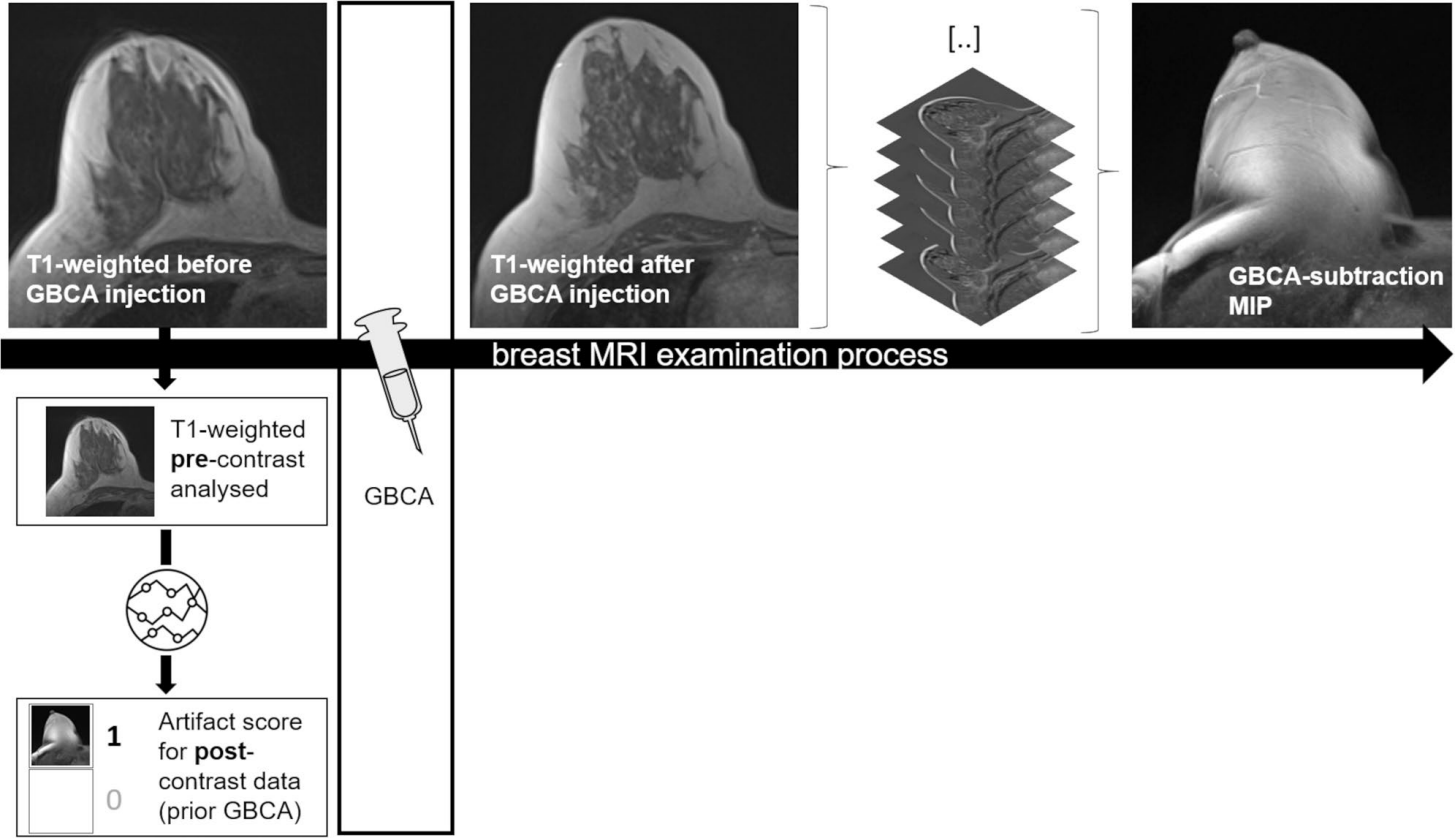
Depiction of the concept for the neural network in not only detecting existing artifacts but also using AI to predict artifacts before they occur. Common examination workflows regarding the dynamic contrast-enhanced acquisitions consist of acquiring pre-contrast T1-weighted sequences, then injecting the gadolinium-based contrast agents (GBCAs) and repeating the T1-weighed acquisition. After the secondary acquisition, the pre- and post-contrast images are subtracted, and maximum intensity projections are created

A 3D-DenseNet201 neural network [16] was trained using as input the unenhanced T1-weighted sequence and as targets the binarized artifact classes described (see Fig. 3). The main training and validation dataset was used in fivefold stratified cross-validation training [17, 18]. The five best-performing models trained during the cross-validation were then combined into an ensemble classifier, which was used to predict artifacts in the holdout test dataset. Details about the choice of the T1-weighted sequence, its preprocessing, the neural network training, hyper-parameters, final model choice, and creation of the ensemble classifier can be found in the Supplementary information.

### Diagnostic performance and statistical analysis

For the evaluation of the performance, receiver operating characteristic (ROC) and precision-recall (PR) curves were used together with their areas under the curve (AuROC and AuPR), sensitivity, specificity, accuracy, F1 score, and positive and negative predictive values (PPV, NPV). Optimization of the positive class probability cutoff was performed to achieve a maximal sensitivity while achieving a specificity of as close as possible to 0.9. To assess the networks’ potential to predict artifacts, class activation maps were computed using the GradCam++  [19] algorithm from the best-performing cross-validation model (as defined by AuROC) and then interpreted by an experienced radiologist. The Chi-squared test was applied to test whether statistically significantly different distributions of artifacts occurred between the different scanner types or between the training and test sets. McNemar tests were performed to determine whether differences in accuracy, sensitivity, or specificity occurred between the ensemble classifiers. For comparing artifact classification between the different readers, a weighted Cohen’s kappa was calculated between each two readers, and a Fleiss kappa was calculated to compare the overall agreement between the raters. The statistical analyses were performed with MATLAB v2020b.

## Results

### Study sample characteristics

After applying the exclusion criteria (Fig. 1), *n* = 2844 breast MRI examinations from *n* = 1982 patients (age: mean 49 ± SD 12.5 years) were included. Among this cohort, breast MRI examinations with significant findings, defined as BI-RADS 3 or higher, were described in *n* = 899 (35.2%) examinations in the training and validation dataset and *n* = 103 of the examinations in the independent test dataset (36.2%). Within the dataset, *n* = 633/2844 (22.3%) breast MRI examinations were acquired on a 1.5-T Magnetom Avanto, along with *n* = 388/2844 (13.7%) on a 1.5-T Magnetom Aera, *n* = 874/2844(30.7%) on a 3-T Magnetom Skyra Fit, and *n* = 943/2844 (33.2%) on a 3-T Magnetom Vida. Details of the cohort and the training and test sets are given in Table 2.

**Table 2:** Overview of the training and validation and the independent test datasets

	Training and validation	Independent test
Examinations	2559	285
Percent	90%	10%
Patients with 1 acquisitions	1388	250
Patients with 2 acquisitions	258	13
Patients with 3 acquisitions	107	3
Patients with 4 acquisitions	49	-
Patients with 5 acquisitions	26	-
Patient with 8 acquisitions	1	-
Age		
Mean	49.6	49.7
SD	± 12.6	± 11.8
BI-RADS scores of 3 and higher		
BI-RADS 3 N/N (%)	177/2559 (6.9%)	22/285 (7.8%)
BI-RADS 4 N/N (%)	258/2559 (10.1%)	33/285 (11.6%)
BI-RADS 5 N/N (%)	127/2559 (5.0%)	15/285 (5.2%)
BI-RADS 6 N/N (%)	337/2559 (13.2%)	33/285 (11.6%)
MRI devices used		
1.5-T Aera N/N (%)	353/2559 (13.8%)	36/285 (12.6%)
1.5-T Avanto N/N (%)	577/2559 (22.5%)	57/285 (20.0%)
3-T Skyra N/N (%)	844/2559 (33.0%)	89/285 (36.1%)
3-T Vida N/N (%)	785/2559 (30.7%)	103/285 (31.2%)

Significant findings were defined as a BI-RADS 3 or higher BI-RADS score given in the radiologist’s reports

*Abbreviations*: *SD*, standard deviation; *MRI*, magnetic resonance imaging

### Frequency of image artifacts on contrast-enhanced subtraction MIPs

The frequency of the Likert-like scale values of image artifacts on the examination level for each of the readers is presented in Table 3. Over the whole dataset, a substantial agreement was achieved for the binarized classes (Fleiss kappa = 0.61), with the agreement in the multiclass classification being slightly lower (Fleiss kappa = 0.41), demonstrating a moderate agreement between the individual three readers for the 5-point artifact classes. Exemplary cases of the different scorings provided are shown in Fig. 2. After applying the majority voting, potentially significant artifacts were identified in *n* = 1521 (53.6%) of the CE subtraction MIPs. The distribution of significant artifacts after binarization in the MRI systems was as follows: Avanto, 1.5 T, *n* = 305/633 (48.2%); Aera, 1.5 T, *n* = 219/388 (56.4%); Skyra Fit, 3 T, *n* = 425/874 (48.6%); Vida, 3 T, *n* = 572/943 (60.1%). In the independent test set, *n* = 155 (54.4%) examinations had a significant artifact. In the training set, *n* = 1366 (53.4%) examinations had significant artifacts. No significant differences in the distribution of the artifacts or systems were found between the training and validation and test datasets (*p* = 1.0).

**Table 3:** Overview of the artifact readings by all individual readers in both the training and validation and the independent test datasets

	Training and validation			Test		
Artifact scores	Reader 1	Reader 2	Reader 3	Reader 1	Reader 2	Reader 3
1 N/N (%)	12/2559 0.5%	82/2559 3.2%	77/2559 3.0%	2/285 0.7%	9/285 3.1%	8/285 2.8%
2 N/N (%)	331/255912.9%	461/2559 18.0%	403/2559 15.7%	40/285 14.0%	48/285 16.8%	41/285 14.4%
3 N/N (%)	634/2559 24.8%	776/2559 30.3%	710/2559 27.7%	61/285 21.4%	91/285 31.9%	85/285 29.8%
4 N/N (%)	711/2559 27.8%	654/2559 25.6%	804/2559 31.4%	81/285 28.4%	72/285 25.2%	95/285 33.3%
5 N/N (%)	871/2559 34.0%	586/2559 22.9%	565/2559 22.1%	101/285 35.43%	65/285 22.8%	56/285 19.6%

Artifact scores were given as follows: *1* no artifact at all; *2* minor artifacts, but without the potential to impede the assessment; *3* moderate artifacts, potentially impairing the visual assessment; *4* pronounced artifacts; *5* highly significant artifacts

### Artifact origin evaluation

**Fig. 4:**
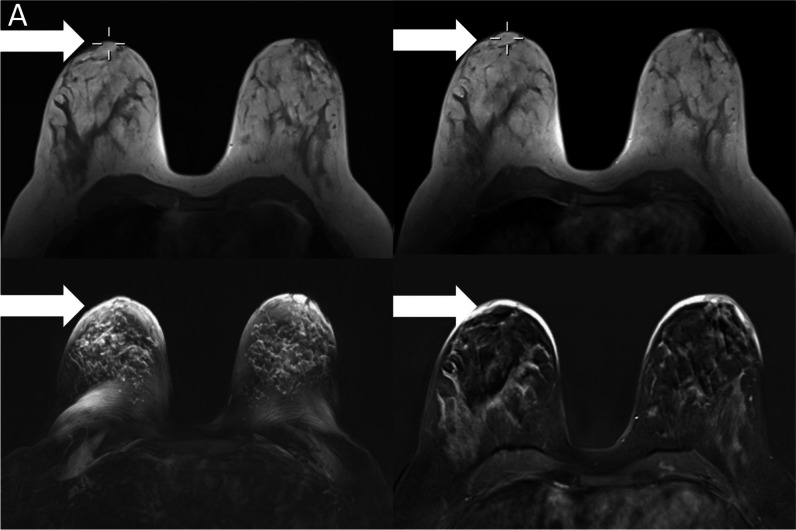

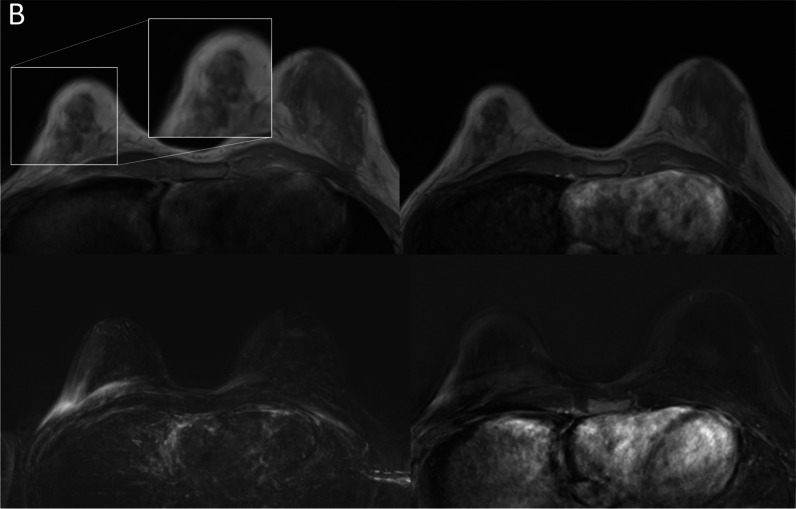

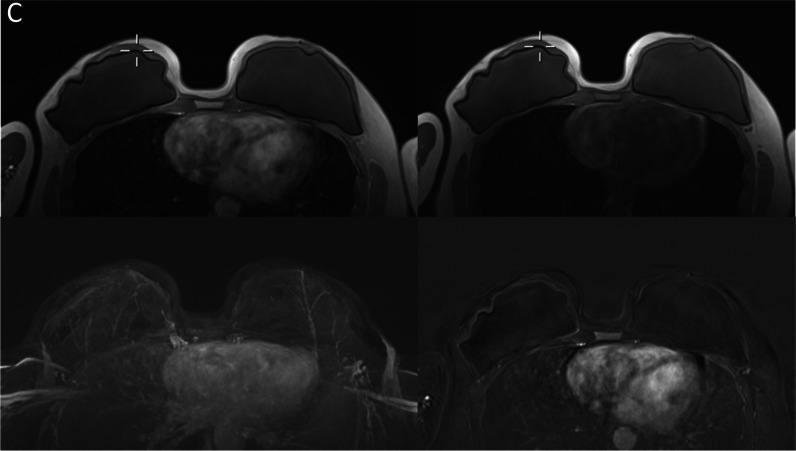
**A** Example case of a patient with significant (static shifting in between acquisitions) artifacts in the subtraction and subtraction maximum intensity projections (MIPs). Upper row demonstrates T1w-unenhanced (left) and T1w-enhanced (right) acquisitions, with the localization cross demonstrating a shift in between the acquisitions. The acquisitions themselves do not show significant blurring, which results in artifacts in the MIP (lower left) and the single-slice subtraction data (lower right). **B** Example case of a patient with significant blurring (movement) artifacts in the subtraction and subtraction maximum intensity projections (MIPs; lower left image). The upper row demonstrates T1w-unenhanced (left) and T1w-enhanced (right) acquisitions, with the magnification demonstrating the movement-related blurring that occurred during the pre-contrast acquisition, resulting in artifacts in the subtraction MIP (lower left) and the single-slice subtraction data (lower right). **C** Example case of implant-associated artifacts. The upper row demonstrates T1w-unenhanced (left) and T1w-enhanced (right) acquisitions, with the subtraction (lower right) demonstrating a subtle linear signal intensity increase around the implant summing up to artifacts in the subtraction maximum intensity projections (lower left)

Figure 4 shows an example of the origin of an artifact based on the retrospective evaluation of T1-weighted acquisition before the GBCA administration. Analysis of the artifact origin revealed the majority of artifacts (39/59, 70%) receiving an artifact score of 4 or 5 on the MIPs to be movement-associated (see Fig. 4a and b). Other types of artifact origins were associated with skin thickening, especially in post-mastectomy cases (9/59, 15%) and women with implants demonstrating subtle enhancing lining around the capsule without fulfilling the criteria for capsule fibrosis (18.6%, 11/59; Fig. 4c).

Subdifferentiation of the movement artifacts, *n* = 39, demonstrated that most of these cases were visible image blurring (Fig. 4a) on either the pre-contrast T1w acquisition, *n* = 14/39 (35.9%), or both the pre- and post-contrast T1w images, *n* = 15/39 (38.5%). The rest of the movement artifacts showed a significant change of the position between the pre- and post-contrast acquisitions (Fig. 4b) without strong blurring in either: *n* = 6/39 (15.3%). In *n* = 4/39 (10.3%), blurring was observed in only the post-contrast image.

### Neural network performance

**Fig. 5:**
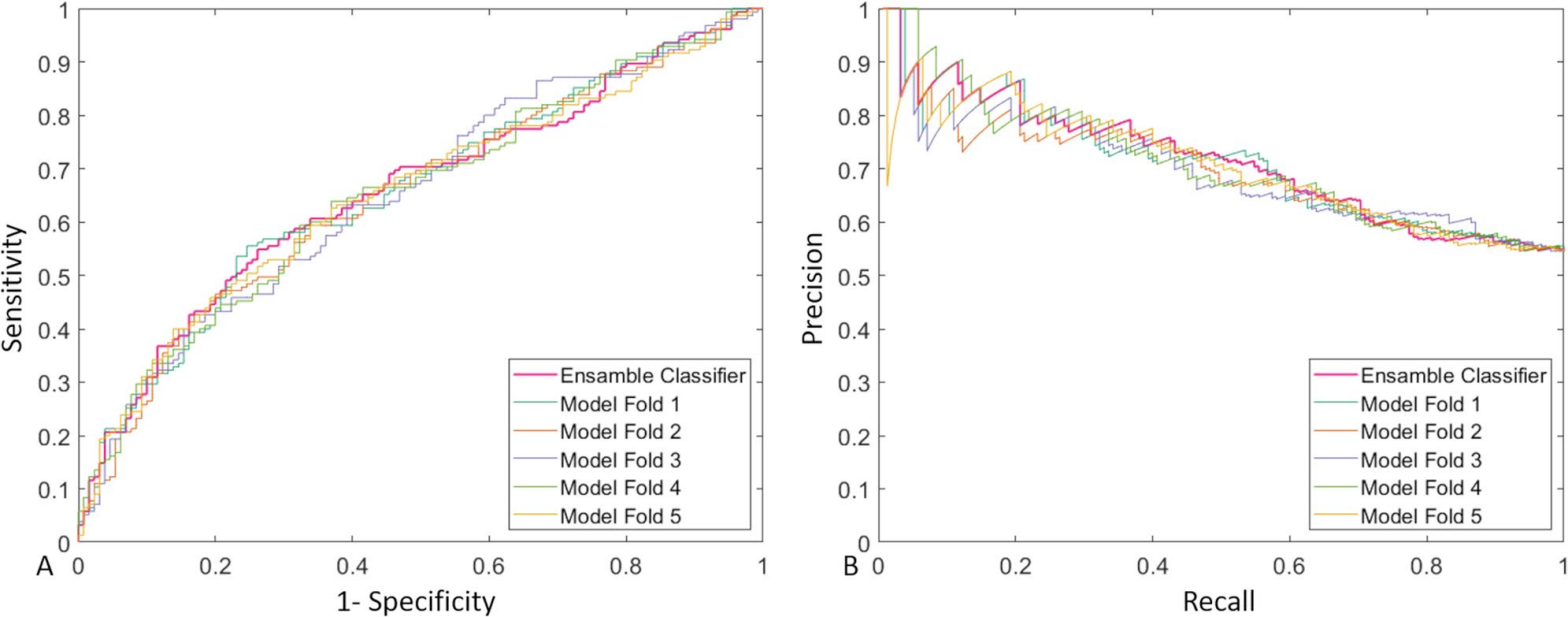
AuROC curve of the ensemble classifier for prediction artifacts in breast MRI contrast-enhanced subtraction images before starting the gadolinium-based contrast agent administration

Using the ensemble classifier, the network achieved an AuROC of 0.66 and an AuPR of 0.71 with the ROC and PR curves in Fig. 5 for predicting the artifacts in the independent holdout test dataset on the CE subtraction images before GBCA administration. At a predefined specificity level of 0.89, it thus achieved a sensitivity of 0.31: able to detect around one in three artifacts in GBCA-subtraction data before starting the contrast agent administration. At the mathematically optimal AuROC cutoff point, the ensemble classifier achieved a sensitivity of 0.59 at a specificity level of 0.62. Detailed performance metrics of each fold and the ensemble classifier on the test set can be found in Table 4.

**Table 4:** Diagnostic performance

	Fold 1	Fold 2	Fold 3	Fold 4	Fold 5	Ensemble classifier	Optimized ensemble classifier
Sensitivity	0.58 (90/155)	0.61 (94/155)	0.59 (91/155)	0.58 (90/155)	0.60 (93/155)	0.59 (92/155)	0.31 (48/155)
Specificity	0.68 (88/130)	0.63 (82/130)	0.62 (80/130)	0.63 (82/130)	0.63 (82/130)	0.62 (80/130)	0.89 (116/130)
NPV	0.58 (88/153)	0.57 (82/143)	0.56 (80/144)	0.56 (82/147)	0.57 (82/144)	0.56 (80/143)	0.52 (116/223)
PPV	0.68 (90/132)	0.66 (94/142)	0.65 (91/141)	0.65 (90/138)	0.66 (93/151)	0.65 (92/142)	0.77 (48/62)
F1	0.63	0.63	0.62	0.61	0.63	0.62	0.44
Accuracy	0.63 (178/285)	0.62 (176/285)	0.60 (171/285)	0.60 (172/285)	0.61 (175/285)	0.60 (172/285)	0.58 (164/285)

For folds 1–5 and for the ensemble classifier, the optimal cutoff point is given with maximized accuracy. For the optimized ensemble classifier, the cutoff point as close as possible to a specificity of 0.9 is given

**Fig. 6:**
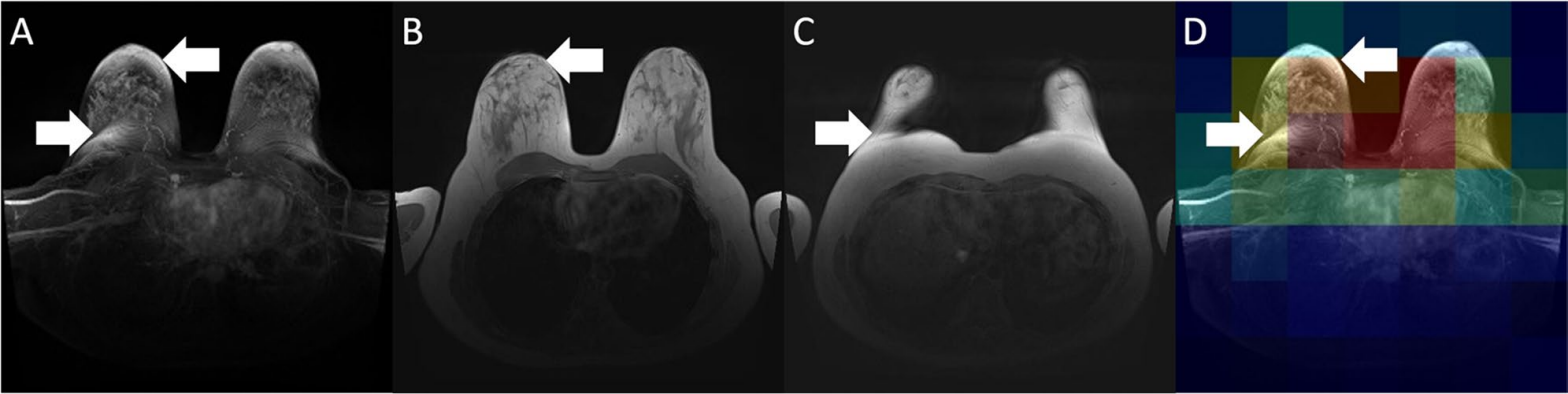
Example of a breast MRI examination with significant artifacts in the subtraction data (**A**), with **B** and **C** representing slices identified as providing subtle movement artifacts (**B**) and field inhomogeneity leading to a higher signal intensity (**C**). **D** demonstrates a GradCAM to illustrate the network to base its predictive decision mostly within the target areas identified in **B** and **C**. Blue areas indicate regions with a low contribution to the final prediction, and areas marked with red indicate regions with a high contribution to the final prediction

An evaluation of the GradCam images can be found in Fig. 6. Notably, the GradCam points to the central part of the volume and to areas close to the regions affected by artifacts in the MIP. These regions correlate to areas affected by motion artifacts (Fig. 6B) or intensity flare-ups (Fig. 6C).

## Discussion

This study demonstrates that predicting the occurrence of artifacts before the GBCA injection seems possible for breast MRI subtraction MIPs. Our results suggest that a neural network can already forecast some artifacts on CE subtraction data before the injection by analyzing the unenhanced T1-weighted series acquired immediately before it. The neural network achieved a moderate AUC of 0.66 for this predictive task and provided a high specificity of 89% while being able to predict about one in three artifacts in the subtraction data.

Providing a reliable and reproducible image quality is an important aim in diagnostic imaging, especially in artifact-prone imaging examinations such as MRI. Identifying potential sources impeding the diagnostic assessment (image artifacts) is thus an important aspect of quality control. Although undoubtedly highly relevant, detecting an artifact in an acquired imaging sequence implies that the formation of this artifact has already occurred. To remove the artifact from the diagnostic assessment, it is thus commonly necessary to repeat the acquisition of the respective image. Therefore, technologies to forecast artifacts are a highly desirable target. This would allow intervening before artifacts occur and thus a true predictive intervention approach for diagnostic imaging processes.

Breast MRI examinations are increasingly evaluated regarding their usability for screening purposes, as either an adjunct or potential complementary screening method in specific cohorts, e.g., women with highly dense breast tissue in X-ray mammography [20–22]. A landmark paper by Kuhl et al in 2014 presented one of the initial studies demonstrating the capability of using an abbreviated breast MRI protocol consisting only of the unenhanced T1-weighted acquisition and a single T1-weighted acquisition after contrast agent administration [3, 4]. Both sequences were subsequently used to derive subtraction series that enabled creating MIPs as the initial reading sequence. Such a subtraction MIP has been suggested to allow assessing the entire breast volume of both sides in a single imaging volume while preserving a high negative predictive value of  > 99% [3]. Several studies have demonstrated a high diagnostic value of this approach, allowing the detection of suspicious findings with a high sensitivity [23–25]. However, breast MRI acquisitions are generally prone to artifacts [6–8]. If subtraction images are generated and further processed to MIPs to facilitate visualization for the radiologist, artifact sources and artifacts present in different slices of the volume might add up to each other. In our study, artifacts scored with the highest severity in the multi-class reading were described in 19–35.4% of the evaluated MIPs by the three readers. This is in the range of artifact rates described by Carbonaro et al (35%) [8], Fiaschetti et al (16%) [9], and Clauser et al (46%) [7].

Developing our approach importantly considered how such technologies might be blended into clinical routine workflow to open up the potential for clinical translation. Unlike many quality-assurance approaches, rather than providing retrospective quality documentation, we aimed to disrupt this principle by developing a true prospectively oriented intervention point for technologists. Thus, we wanted to modify the course of an examination before the occurrence of an image artifact. Forecasting an artifact likelihood for “yet-to-start” parts of the MRI protocol based on analyzing the already acquired part of the examination provides such a prospective intervention point. With dynamic GBCA-enhanced acquisitions commonly not being repeatable once the GBCA is injected, we further focused on a step that is thus especially clinically critical during the examination. Therefore, the multiclass reading was transformed into a binarized label for further analysis. This allowed the algorithm to provide a clear recommendation to intervene before starting the GBCA administration or continuing with the examination. However, just providing visual scorings of the expected artifact likelihood before the GBCA administration could pose a reasonable alternative, leaving the decision on how to proceed more open to the technician.

Overall, the network achieved a moderate but not outstanding performance compared to classical post-acquisition artifact detection algorithms working on data already acquired. However, this might be seen as a strength rather than a weakness of the study. Artifacts that are predictable in the near future based on the non-CE T1-weighted acquisitions before the GBCA administration must be somehow represented in the data analyzed. This cannot account for all artifacts visible on the subtraction images, since artifacts with the source occurring after GBCA administration, e.g., due to patient movement with GBCA inflow [8], inevitably cannot be predicted by analyzing the T1-weighted sequence before GBCA injection if the patient did not move. This obviousness also correlates to our data indicating that movement artifacts were the most challenging for the algorithm compared to artifacts of other origins. Providing artifact sources as labels to the technician could further stratify interventions. For example, we observed some artifacts originating from subtle enhancements around breast implants (not fulfilling capsule fibrosis criteria). Even if predicted correctly, such artifacts could not be avoided by current interventions since they originate from biological tissue behavior and thus might not necessarily be reported during the scan. Still, the neural network predicted many artifacts in the dataset, indicating the clinical potential of such an intervention point while preserving a high specificity to avoid too many unnecessary calls.

Thus, the technique described here in a first feasibility study might have highly significant clinical implications for breast MRI. It could allow work in the routine workflow and still improve breast MRI by not only documenting when something went wrong, but also helping to avoid future obstacles that can be anticipated during an ongoing examination. In case of predicting a high likelihood of an artifact in the upcoming part of the examination, the technician could thus intervene before starting GBCA injection, such as by advising the patient to lie still or using a less movement-prone acquisition sequence. Such information given to the patient has been suggested to positively influence artifact occurrence, even if provided before the scan [26]. However, these interventions must be balanced against prolonging an MRI examination with the interventions because longer examination times might themselves increase the risk of motion artifacts [27].

Our study has several notable limitations. First, the sample size is relatively small, with only *n* = 2844 breast MRI datasets. Although this is one of the largest studies in the field of breast MRI artifact assessments, future studies should examine the generalizability of the approach regarding different vendors and various settings, such as different breast coils. In addition, different T1-weighted image acquisition techniques should be investigated to assess the generalizability of different acquisition techniques used in GBCA-enhanced imaging. Another limitation is that only three readers performed the artifact reading, and a larger number is desirable in future studies. Although we demonstrated the neural network’s ability to predict artifacts before they occurred, we did not conduct a prospective study to investigate the clinical impact. Therefore, we cannot yet determine the degree to which the predicted artifacts would have been preventable by an intervention or the overall reduction rate of artifacts in abbreviated breast MRI protocols achieved with the neural network in a clinical setting. Also included in this limitation is that we considered any artifacts detected, independent of their factual significance in impeding the diagnostic assessment for the full-breast MRI examination in the respective case. As such, we did not investigate whether an existing lesion was obscured in the individual cases, so the presence of an artifact must not have necessarily resulted in a non-diagnostic breast MRI examination in our retrospective dataset. Thus, the clinical applicability and impact cannot be derived from our data alone. Future studies are necessary to further investigate this predictive approach for MR artifacts and determine the robustness and generalizability of the approach. Despite these limitations, the sensitivity of the neural network may be sufficient to significantly improve workflow efficiency in large-scale settings, such as screening, by avoiding many repeat examinations.

In conclusion, our results demonstrate the technical feasibility of using a neural network to predict some artifacts that emerge in GBCA-enhanced breast MRI before GBCA administration. If confirmed in larger studies, this raises the possibility of developing intervention strategies to further advance GBCA-enhanced breast MRI by reducing image artifacts. Further research is necessary to develop and evaluate such intervention strategies.

### Supplementary Information

Below is the link to the electronic supplementary material.

Supplementary file1 (PDF 288 KB)

## Abbreviations

AuPR: Area under precision-recall curve
AuROC: Area under receiver operating characteristic curve
CE: Contrast-enhanced
CNN: Convolutional neural network
DCE: Dynamic contrast-enhanced
DWI: Diffusion-weighted imaging
EC: Ensemble classifier
GBCA: Gadolinium-based contrast agent
MIP: Maximum intensity projection

## Declarations

### Guarantor

The scientific guarantor of this publication is Andrzej Liebert.

### Statistics and biometry

Lorenz A. Kapsner and Andrzej Liebert have significant statistical expertise.

### Study subjects or cohorts overlap

A total of 2265 subjects have been previously reported in “Automated artifact detection in abbreviated dynamic contrast-enhanced (DCE) MRI-derived maximum intensity projections (MIPs) of the breast.” Eur Radiol. https://doi.org/10.1007/s00330-022–08626-5.

A total of 1309 subjects have been previously reported in “Image quality assessment using deep learning in high b-value diffusion-weighted breast MRI.” Kapsner, L.A., Balbach, E.L., Folle, L. et al Image quality assessment using deep learning in high b-value diffusion-weighted breast MRI. Sci Rep 13, 10549 (2023). https://doi.org/10.1038/s41598-023-37342-3

### Methodology

• retrospective

• diagnostic or prognostic study

• performed at one institution
