# Supplementary material for: Smart forecasting of artifacts in contrast-enhanced breast MRI before contrast agent administration
Source: Eur Radiol. 2023 Dec 15;34(7):4752–63. doi: 10.1007/s00330-023-10469-7 (PMC11213750; doi:10.1007/s00330-023-10469-7)
Supplement: Supplementary file 1 — Supplementary file1 (PDF 288 KB) [file 330_2023_10469_MOESM1_ESM.pdf]

Smart forecasting of artifacts in contrast enhanced breast MRI before contrast agent administration

Electronic Supplementary Material (ESM)

Eur Radiol (2023) Liebert A, Das BK, Kapsner LA et al.

Material and Methods:

Image processing for artifact evaluation

All data were transferred to scientific research workstations, allowing for further processing and the analysis of the data. The pre-contrast T1-weighted acquisition and the second post-contrast dynamic T1-weighted acquisition (about 120sec after intravenous application of GBCA) were extracted from the multiparametric protocol to be used for further steps in the study. The second time point after GBCA administration was chosen as reflecting the sequence routinely used for assessing the presence of lesions in subtraction MIPs in our hospital [1].

Using this data, contrast enhanced subtraction series were created by subtracting the pre-contrast unenhanced T1-weighted sequence from the second postcontrast T1-weighted sequences.

All subtraction images of an individual series were then transformed into a single image maximum intensity projection (MIPs) using an in-house developed Python (version 3.9.13) code in order to represent the voxels with the highest intensity values along the z-axis on a 2D-image as previously described in our own work [1]. The resulting images were divided into two halves, representing the left and right breast in order to be used as an independent target region of interest (ROI) for further visual and technical evaluation.

Input data preprocessing:

The study aimed to investigate whether artifacts visible on contrast enhanced subtraction MIPs can be predicted in advance, before the GBCA-administration has started. Thus, we chose as inputs for the neural network the unenhanced T1-weighted sequence. This sequence is acquired directly before starting the process of the intravenous injection of the GBCA in the patient and thus allows to assess whether artifacts emerging after the GBCA-injection might be truly predictable. In order to homogenize the resolution of the data, each of the T1-weigthed volumes was resampled before the input into the neural network to a matrix size of 224x224x56. Additionally, the intensities of the T1-volume were scaled to a unit range using the minimum and maximum intensities over the whole volume.

Neural Network Architecture

A 3D-DenseNet 201 [2] Neural network was implemented in Python (version 3.9.13) based on the MONAI (version 0.8) [3] frameworks implementation in PyTorch (version 1.10.2). No pretraining of the networks model was used as the network as implemented as a 3D network. The network was trained in a five-fold cross validation training in which the data of the training/validation dataset was further split into a training dataset and a validation data set with approximately 80% and 20% of the whole training/validation dataset (n=2559 MRI examinations). The training was performed using a learning rate of 10^{-7} and a batch size of 16. The samples in each batch were drawn randomly using a weighted random sampler in which each of the samples were given a weight based on the prevalence of its binary label. The training was performed using cross entropy loss. The network was trained for 500 epochs without early stopping reaching the lowest value of the validation loss in epoch: 278, 264, 306, 221 and 266 for the folds 1 to 5 respectively. Changes of the training loss and of the validation loss during training are presented in Supplement Figure 1.

Ensemble classifier

From each of the five cross-validation folds, the weights from the epoch with the lowest validation loss, observed within 500 epochs, were chosen to create five individual ‘final’ cross-validation models. These five individual models were then combined into an ensemble classifier to predict artifacts in the holdout test dataset by calculating the mean of the prediction probability from all five models for both classes.

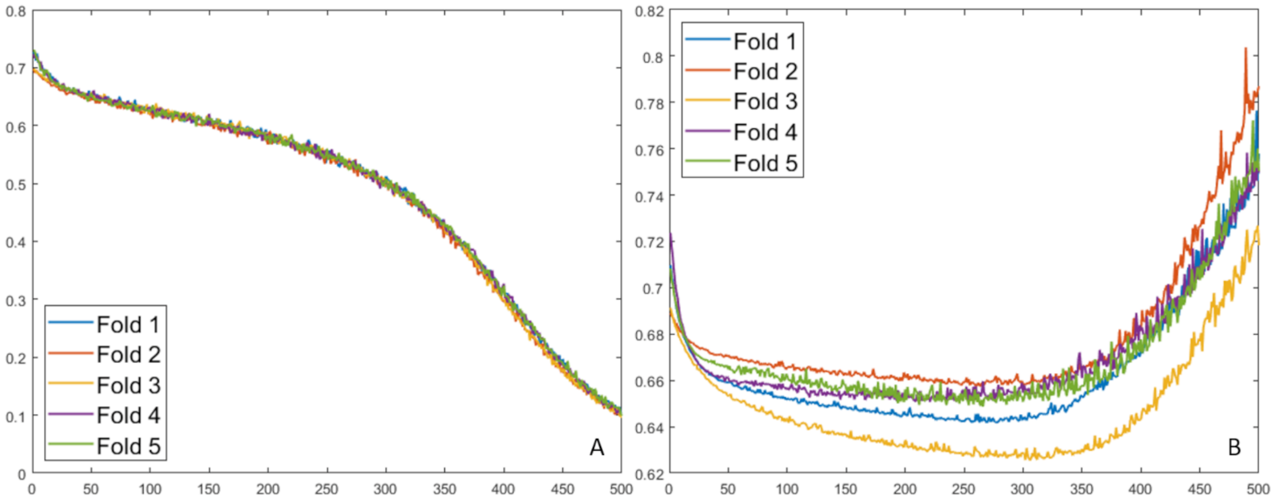

Supplement Figure 1: Change of the training loss (A) and of the validation loss (B) for each of the five folds during the cross-validation training.

References

1 Kapsner LA, Ohlmeyer S, Folle L et al (2022) Automated artifact detection in abbreviated dynamic contrast-enhanced (DCE) MRI-derived maximum intensity projections (MIPs) of the breast. Eur Radiol. 10.1007/s00330-022-08626-5

2 Huang G, Liu Z, Van Der Maaten L, Weinberger KQ (2017) Densely connected convolutional networksProceedings of the IEEE conference on computer vision and pattern recognition, pp 4700-4708

3 Cardoso MJ, Li W, Brown R et al (2022) MONAI: An open-source framework for deep learning in healthcare. arXiv preprint arXiv:221102701
